# Leading From Higher Headquarters

**DOI:** 10.1111/sjop.13120

**Published:** 2025-04-30

**Authors:** Jostein Mattingsdal, Roar Espevik

**Affiliations:** ^1^ Royal Norwegian Naval Academy, Leadership Division Norwegian Defense University College Laksevåg Norway; ^2^ Center for Crisis Psychology, Faculty of Psychology University of Bergen Bergen Norway

**Keywords:** collaborative crisis response, decision‐making performance, goal setting, indirect leadership, self‐efficacy

## Abstract

This study explores the relationship between self‐efficacy, task acceptance, and goal setting among senior leaders operating in higher headquarters, particularly within the context of hybrid warfare. The aim is to describe the factors that influence the decision‐making (DM) performance of police and military leaders involved in collaborative response efforts during wartime. Path analysis was utilized to investigate an original dataset comprising 102 participants from Norway's police and military (*M* = 44.4 years). It involved a realistic simulation exercise designed to replicate the challenges associated with hybrid warfare. The study was grounded in Bandura's Social Cognitive Theory. The results indicated that self‐efficacy demonstrated a positive and significant indirect effect on DM performance through goal setting. Furthermore, high DM performance in peacetime and high goal setting were both positively associated with DM performance in wartime. This study enhances the understanding of the interplay between self‐efficacy, task acceptance, and goal setting in crisis response settings. The findings underscore the significance of cultivating effective DM skills in senior leaders operating in higher headquarters, particularly in defense against hybrid warfare. These insights can be applied to leadership development and policy programs aimed at enhancing national DM frameworks.

**Summary**

- Self‐efficacy: Indirectly improves wartime decision‐making by encouraging ambitious goals; no direct effect.
- Peacetime Preparation: Strong peacetime decision‐making and proactive goal setting lead to better wartime outcomes.
- Hybrid Warfare: Ambiguity reduces the standalone value of individual self‐efficacy, highlighting the need for teamwork.
- Policy Recommendations: Focus on adaptive leadership simulations, interagency collaboration, and mentorship to enhance national crisis readiness.

## 1 Introduction

In Western countries, government officials are finding it increasingly difficult to manage security crises effectively (Stein 2023). As global competition intensifies, the nature of threats has become harder to define (Mumford and Carlucci 2023). Traditional crisis response methods, such as straightforward military or police actions, may no longer suffice for new and emerging threats (Borch and Heier 2024). This places high demands on leaders engaged in crisis response to determine the appropriate ends, ways, and means.

One major shift in the contemporary security landscape is the rise of hybrid warfare, which combines conventional military actions, unconventional tactics, cyber‐attacks, and information manipulation (Weissmann et al. 2021). This new type of warfare presents unique challenges for leaders (Caliskan and Liégeois 2021). An example of hybrid warfare is Russia's efforts to destabilize NATO countries, which exploit weaknesses in information sharing and public trust (Wilson and Cadin 2024). This has become a key focus for security experts and policymakers (Bankov 2024).

As a NATO member bordering Russia and a major energy supplier to Europe, Norway faces unique security challenges (Wegge 2023). Its critical infrastructure, such as the energy grid and transportation systems, is vulnerable to hybrid warfare (Østensen and Bukkvoll 2018). Additionally, the complex relationship between the Norwegian police and military can hinder their response to these threats (Auglend 2015). This makes Norway's higher headquarters a crucial setting for investigating effective decision‐making (DM) frameworks against hybrid warfare.

### 1.1 Research Focus and Theoretical Framework

This study aims to investigate how the self‐efficacy, goal setting, task acceptance, and peacetime DM performance of Norwegian police and military leaders predict their DM performance in wartime. Based on Bandura's (2023) Social Cognitive Theory (SCT), we conceptualize leaders as proactive agents who integrate prior experiences (e.g., international combat operations) and real‐time stakeholder input (e.g., civilian collaborators) to navigate crises. For example, a military leader may adapt NATO‐derived tactics to domestic constraints during a hostage situation, balancing prior training with local resource realities.

#### 1.1.1 Core Mechanisms

Leaders with high self‐efficacy are more likely to accept complex tasks and set quantifiable performance goals (e.g., “neutralize threat in Sector X within 90 min”), thereby fostering accountability and strategic focus (Bandura 1997; Locke and Latham 2013). A feedback loop is created: achieving goals reinforces self‐efficacy, which in turn motivates higher‐order achievements (Bandura and Wood 1989). While specific performance goals can enhance DM outcomes (Taylor et al. 2011), overly rigid targets may stifle innovation, a tension that can be mitigated through iterative feedback and ethical prioritization (Swann et al. 2021; Gray 2015).

Moreover, operational leaders operationalize performance goals through frameworks such as NATO directives (2019), aligning tactical actions with organizational standards (e.g., non‐lethal engagement protocols), while retaining flexibility for emerging threats (Mattingsdal, Aandal, et al. 2023; Mattingsdal, Johnsen, et al. 2023; Rutherford and Meier 2015). As outlined in SCT, this process entails continuous monitoring, evaluation, and possible revision based on how an individual's performance aligns with assigned goals and perceived progress. This emphasizes the fluid nature of DM as it adapts to new insights regarding emerging tasks.

The inherent need for continuous situational assessments and goal recalibration highlights leaders' cognitive readiness despite ambiguity (Grier 2012; Senko and Dawson 2017; Zhang et al. 2023). Additionally, successful leaders must reconcile mission accomplishments with ethical constraints (e.g., civilian safety), thereby avoiding reactive compromises (Chiriac 2022; Dwyer 2019). To navigate these complexities during operational duties, effective leadership depends on the efficacy beliefs of individual decision‐makers, enabling them to make prudent choices rather than impulsive ones (Johnsen et al. 2017).

This framework advances recent research on DM by operational leaders (Mattingsdal et al. 2024) by quantifying how personal factors jointly shape crisis leadership.

### 1.2 Research Question

Effective leadership and interagency cooperation are critical components of addressing modern security threats (Storberget et al. 2023). Understanding how police and military leaders are influenced by personal, behavioral, and environmental factors is essential for ensuring that they are interoperable when working together. By examining the complex interplay between efficacy beliefs and DM in the context of hybrid warfare, our study sought to provide valuable insights into the challenges of responding to new and emerging security threats. The study focused on the following research question:

How do self‐efficacy, peacetime DM performance, goal setting, and task acceptance impact the DM performance of senior leaders during war?

#### 1.2.1 DM Performance Evaluation

Two high‐ranking subject matter experts (SMEs), one police and one military officer with extensive operational experience in crisis contexts, independently assessed participants' decisions. Blinded to the study's objectives, the SMEs evaluated scenario‐specific decision feasibility and strategic appropriateness using standardized criteria aligned with the rules of law. This included proportionality (avoiding excessive harm), necessity (limiting actions to those essential for legitimate objectives), distinction (clearly separating combatants from non‐combatants), humane treatment (ensuring individuals are treated humanely), and accountability (adherence to ethical standards). The SMEs were selected for their analytical rigor, cross‐sectoral expertise, and leadership roles in high‐stakes DM environments.

To ensure reliability, we implemented several strategies. First, cross‐validation among SMEs using evaluation criteria that were mission‐anchored minimized individual experiential biases. Second, a two‐expert design was employed to prioritize depth over breadth, allowing for nuanced deliberation, while effectively managing resource constraints like time and budget. Third, relying solely on two SMEs helped maintain evaluation consistency by excluding potential confounding variables, which could lead to asymmetrical interpretations of performance. This approach enhanced ecological validity for assessing leadership‐level DM without sacrificing methodological rigor.

In this context, “War DM performance” refers to the participants' decisions in the simulation's war phase. “Peace DM performance” pertains to the participants' decisions in the peace phase and serves as a comparative benchmark for their DM performance in wartime. It is worth noting that the SME's focused on our participants´ decisions, rather than their subordinates, and therefore did not include subordinates' experiences in the DM performance variable.

### 1.3 Hypotheses

#### 1.3.1 Self‐Efficacy and Wartime DM Performance

The theoretical foundation for our first Hypothesis 1 is based on SCT, which posits that individuals' beliefs in their ability to succeed in specific situations significantly influence their behavior and performance (Stajkovic and Sergent 2019). Individuals with high self‐efficacy tend to set higher goals, commit to them, seek and interpret feedback effectively, and adjust their strategies as needed.

In this study, self‐efficacy specifically refers to participants' beliefs in their capacity to manage crisis response efforts, such as coordinating emergency services, communicating clearly with the public, and making quick, informed decisions during emergencies. Participants with high self‐efficacy should be more likely to adopt leadership styles that emphasize teamwork, develop effective contingency plans, and demonstrate prudent DM in the face of obstacles (Paglis 2010). This concept seems especially important in ambiguous situations involving events that are not easily predictable, making self‐efficacy even more crucial.

Meta‐analyses have demonstrated that self‐efficacy is positively related to various types of performance across multiple domains (Stajkovic et al. 2009). This relationship between self‐efficacy and performance is supported by empirical evidence showing that self‐efficacy influences goal setting, self‐regulation, and the use of analytic strategies (Bandura and Wood 1989). Research has also shown that self‐efficacy influences DM in various domains, including business, education, sports, and healthcare (Mata et al. 2021; Moritz et al. 2000; Schunk and DiBenedetto 2021). In the work‐related tasks of professionals, self‐efficacy has been linked to favorable outcomes, including improved DM (Judge et al. 2007), increased adaptability (Zimmer‐Gembeck 2021), enhanced resilience (Benight and Cieslak 2011), and leadership (Dwyer 2019), and leaders with high self‐efficacy are more likely to demonstrate effective leadership and inspire their teams to perform at their best (Nordmo et al. 2022).

In ambiguous settings such as hybrid warfare, self‐efficacy is particularly relevant (Wang and Hsu 2014). Leaders' beliefs in their ability to navigate the complexities of security threats are essential for effective DM (Fosse et al. 2015). Research has shown that leaders with high self‐efficacy are more likely to take the initiative, exhibit job satisfaction, and demonstrate astute actions (Johnsen et al. 2017). Research specific to military settings highlights that leaders with high self‐efficacy are more likely to exhibit decisive leadership, adapt to changing circumstances, and maintain a high level of performance under pressure (McLarnon et al. 2021).

These findings suggest that self‐efficacy may serve as a critical factor influencing DM performance in higher headquarters during wartime, where the nature of DM is complex and multifaceted (Jervis 2017, p.xvi). Therefore, we propose:

Hypothesis 1 *High self‐efficacy is positively associated with decision‐making performance in war*.

#### 1.3.2 Goal Setting and Wartime DM Performance

Our study focuses on performance goals, quantifiable outcome targets (e.g., arrest suspect A within 60 min), as distinct from learning goals oriented toward skill development. Grounded in goal‐setting theory (Locke and Latham 2013), performance goals enhance motivation, accountability, and tactical focus by providing clear benchmarks for individuals and teams. While excessively rigid goals risk counterproductivity (Swann et al. 2021), well‐calibrated targets improve DM performance in volatile contexts by aligning effort with strategic priorities (Gray 2015; Taylor et al. 2011).

In crisis response operations, performance goals enable leaders to mitigate uncertainty by navigating the ambiguity of threats through measurable endpoints (Bearman et al. 2023). These goals also optimize resource allocation by prioritizing missions, such as hostage rescue versus perimeter defense, based on predefined success metrics. Additionally, they help manage irreversible risks by balancing tactical aggression with restraint through established rules of engagement (Marchau et al. 2019).

Leadership plays a critical role in the effective application of performance goals (Ibrahim and Daniel 2019). Leaders must contextualize challenges by setting goals that are ambitious yet achievable to avoid demoralization (Bong 2001). They should prefer collective goals to foster team cohesion in interdependent tasks (Kleingeld et al. 2011) while mitigating the risk of free riders through individual accountability (Masal and Vogel 2016). Additionally, leaders need to adapt dynamically by shifting toward learning goals during skill gaps, as rigid performance targets may hinder innovation (Seijts and Latham 2012).

In this study, we focused on how participants set performance goals based on anticipated tactical outcomes for upcoming missions. Thus, we propose:

Hypothesis 2 *Setting high performance goals is positively associated with decision‐making performance in war*.

#### 1.3.3 Integrating Self‐Efficacy, Goal Setting, and Task Acceptance in Wartime DM Performance

Building on self‐efficacy and goal setting as cognitive motivators of DM performance in war, we position task acceptance, defined as an individual's willingness to undertake challenging, externally assigned tasks in hierarchical contexts, as a critical enhancer of this relationship. As described by SCT, we theoretically propose that task acceptance, when synergistically paired with self‐efficacy and ambitious goals, promotes critical engagement in ambiguous contexts. This triad facilitates perceived controllability and informed DM by encouraging deliberate problem‐solving even under the urgencies of war (Wood and Bandura 1989, 374).

#### 1.3.4 Self‐Efficacy and Goal Setting

Research has consistently shown that individuals with higher self‐efficacy are more likely to engage in effective goal‐setting behaviors (Huang 2016). Specifically, individuals with high self‐efficacy tend to set more challenging and specific goals, which are essential for achieving success in high‐pressure environments (Margolis and McCabe 2006). This is particularly relevant in military operations, where the ability to set realistic yet ambitious performance goals can significantly impact mission success (Shortland et al. 2019).

#### 1.3.5 Task Acceptance, Self‐Efficacy, and Goal Setting

Task acceptance, the willingness to engage with externally assigned challenges, reflects an individual's anticipatory assessment of their capability to execute those tasks, a core dimension of self‐efficacy (Bandura 1999). Elevated self‐efficacy not only strengthens task acceptance but also drives adaptive goal setting, creating a cascade that enhances DM performance (Bandura 1997). This process is contingent on individuals' perceptions of situational realism and controllability, which are essential for meaningful goal pursuit. Without these personal assessments, goal setting can become disconnected from actionable intent (Locke 2023). As described by Gilbert and Kelloway (2014), leaders who accept the purpose of their mission focus on contextually relevant, agile goal setting, enabling them to effectively address immediate challenges of their organization. Additionally, studies have examined how external factors, such as team dynamics and organizational support, influence team performance (André et al. 2014). This interdependence between personal, behavioral, and environmental factors generates a self‐sustaining cycle; accomplishments boost self‐efficacy, which in turn encourages the pursuit of tasks and increasingly ambitious performance goals (Schunk and DiBenedetto 2020).

Within high‐stakes environments like hybrid warfare, we posit that task acceptance and goal setting act as sequential mediators bridging self‐efficacy and DM performance in war. This leads to:

Hypothesis 3 *Self‐efficacy has a positive indirect effect on wartime decision‐making performance, mediated sequentially by task acceptance and goal setting*.

**FIGURE 1:**
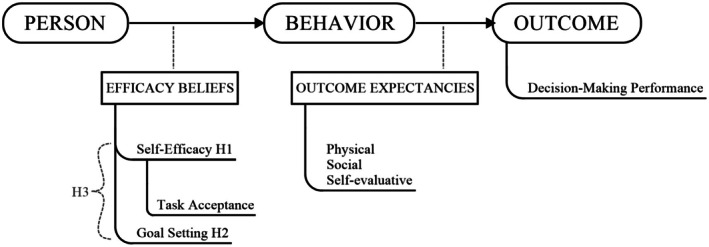
Schematic representation of the hypotheses.

Our hypotheses are illustrated in Figure 1.

## 2 Method

### 2.1 Participants

The participants consisted of 102 high‐ranking officials from Norway's police and military sector (88 males and 14 females). The selection of participants was based on their positions within their respective organizations, ensuring that individuals with significant experience and authority in crisis management were included. The sample comprised 59 military officials (mean age = 44 years; range = 31–58; employment 8–39 years) and 43 police officials (mean age = 45 years; range = 29–56; employment 6–35 years), allowing for a balanced representation of both agencies.

### 2.2 Simulation Exercise

**FIGURE 2:**
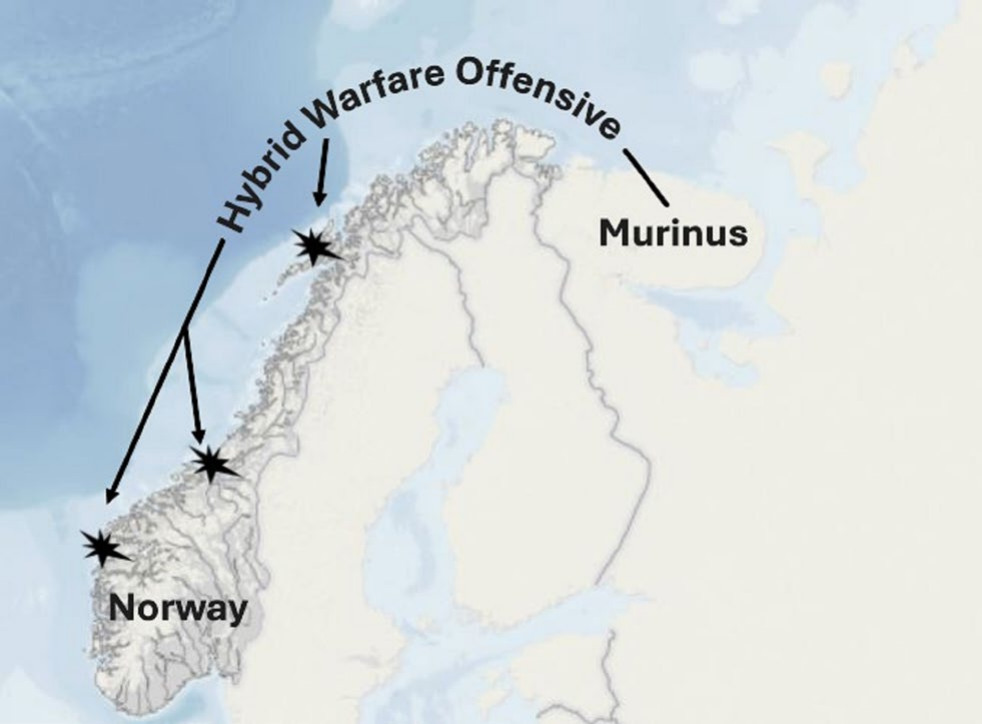
Scenario: In 2026, the fictitious state Murinus faces internal protests over electoral malpractice and blames NATO. Murinus disrupts Norwegian infrastructure and eventually launches an offensive. The image was created by the authors.

The core of the research involved an immersive simulation exercise designed to mimic the complexities of a hybrid warfare scenario (Figure 2). This simulation was based on an unclassified NATO exercise module (Weaver 2021) to ensure relevance. The simulation involved crisis escalation from normalcy to armed conflict, unfolding over 36 missions divided equally into two phases: peace and war.

### 2.3 Procedure

The study's background information and scenario were emailed to the participants prior to the simulation day. The purpose of the study was presented as follows: To assess collaborative crisis response in higher headquarters. The participants were informed that their responses would be anonymized, that they could withdraw at any time, that once they signed the informed consent form, and that communication between them and the researcher would not be allowed once the simulation began. All data was collected electronically. No violations of normality were identified, and there was no missing data.

At the beginning of the simulation, the participants received a scenario update that included strategic guidance and policy instructions. Subsequently, the peace phase of the scenario unfolded, where the participants were presented with 18 mission vignettes that involved three decision tasks:

1. Assigning troops: Participants selected from various police and military resources displayed as multiple‐choice options to form a unit for a specific mission.
2. Guidance on use of force: Participants indicated their instructions on how much force should be used for each mission.
3. Prioritizing missions: Participants indicated the urgency of each mission.

At the end of the peace phase, participants first completed the questionnaire assessing self‐efficacy, task acceptance, and goal setting. A simulated “royal decree” then triggered the transition to war, introducing 18 mission vignettes mirroring peacetime decision tasks but recontextualized for wartime scenarios. Participants retained the option to reject missions, reflecting real‐world operational discretion.

Each phase (peace/war) lasted 30 min, with high‐fidelity vignettes, including realistic tactical briefings, time pressure, and resource constraints, ensuring immersion. To mitigate confounders like attention decay (Dahlstrom et al. 2009), session duration was calibrated to mirror typical headquarters operational tempos, while mission sequencing intended to avoid participant fatigue (Galesic and Bosnjak 2009). This design enabled controlled observation of how personal factors (e.g., self‐efficacy) and contextual shifts (peace → war) interact to shape decisions.

### 2.4 Data Collection and Variables

#### 2.4.1 Endogenous Variables

##### 2.4.1.1 Self‐Efficacy

A Self‐Efficacy Scale, developed following Bandura's (2006) guidelines, was utilized to measure participants' beliefs in their capabilities to handle hybrid warfare. This measure consisted of four items and employed Visual Digital Scales (VDS) ranging from 0 to 100 to gauge both the strength and level of self‐efficacy during war. For analysis purposes, the self‐efficacy variable was categorized into three groups based on equal percentiles of the scanned cases: Low (0–33rd percentile), Moderate (34–66th percentile), and High (67–100th percentile). The Cronbach's alpha for the self‐efficacy scale was 0.87, indicating sufficient consistency and confirming the reliability of the measures.

##### 2.4.1.2 Task Acceptance

Task acceptance was measured after the self‐efficacy variable. A continuous VDS was used to assess how participants accepted guidance from higher headquarters in the war phase. The scale ranged from “very low” to “very high”, with higher scores indicating greater acceptance of higher headquarters' guidance.

##### 2.4.1.3 Goal Setting

Goal setting was measured after the task acceptance variable. To assess the participants goal setting, a continuous VDS was used to measure the level of performance goals established during wartime. The scale ranged from “very low” to “very high,” with higher scores indicating more ambitious goals. For instance, a high goal indicated that the participants aimed to meet all mission objectives, including having no casualties on their side, successfully defeating all enemy forces, and preventing the enemy from achieving any of their aims. To facilitate analysis, the goal‐setting variable was categorized into three bins (low/moderate/high) based on equal percentiles of the scanned cases.

Task acceptance and goal setting were assessed via single‐item continuous VDS, a pragmatic approach aligned with precedent research on complex DM environments (Wood et al. 1990 for goals setting; Hettiachchi et al. 2020 for task acceptance). The Goal Commitment Scale (Hollenbeck and Klein 1987) was excluded due to its focus on post hoc adherence to predefined objectives rather than the content and aims of wartime (e.g., “neutralize enemy within 60 minutes”). While single‐item measures preclude conventional reliability metrics, their use is methodologically justified to minimize respondent burden, when constructs are narrowly defined (Fisher et al. 2016) and contextual specificity outweighs multidimensional assessment (e.g., hostile acts requiring rapid, clear responses).

##### 2.4.1.4 War DM Performance

Two SMEs assessed the participants' DM across the 18 wartime missions. They used the decision tasks to evaluate the participants' leadership abilities through the three decision tasks: (1) Assigning troops: This tested how participants utilized troops on the missions. (2) Guidance on use of force: This assessed the appropriate response level based on the mission's context. (3) Prioritizing missions: This examined how participants judged the missions´ importance. These elements are core strategic assessments that leaders in higher headquarters must evaluate (NATO 2019), helping SMEs understand how effectively participants perceived and managed each aspect.

To quantify the DM performance variable, the SMEs used a 5‐point scale ranging from “poor” to “excellent.” The scores from these assessments were aggregated to generate an overall DM performance score, which was calculated as the mean for each participant. A higher score indicated that a participant was providing appropriate guidance to their subordinates. To ensure fairness and accuracy, the SMEs followed a standardized evaluation framework.

### 2.5 Exogenous Variables

#### 2.5.1 Peace DM Performance

*Peace DM performance* was assessed based on participants' DM during the initial 18 missions in the scenario's peace phase. The SMEs used the same criteria as in wartime to evaluate performance: how participants assigned troops, how they decided on the use of force, and how they prioritized missions. This consistent evaluation method allowed for comparison between peace and wartime DM.

#### 2.5.2 Group

**FIGURE 3:**
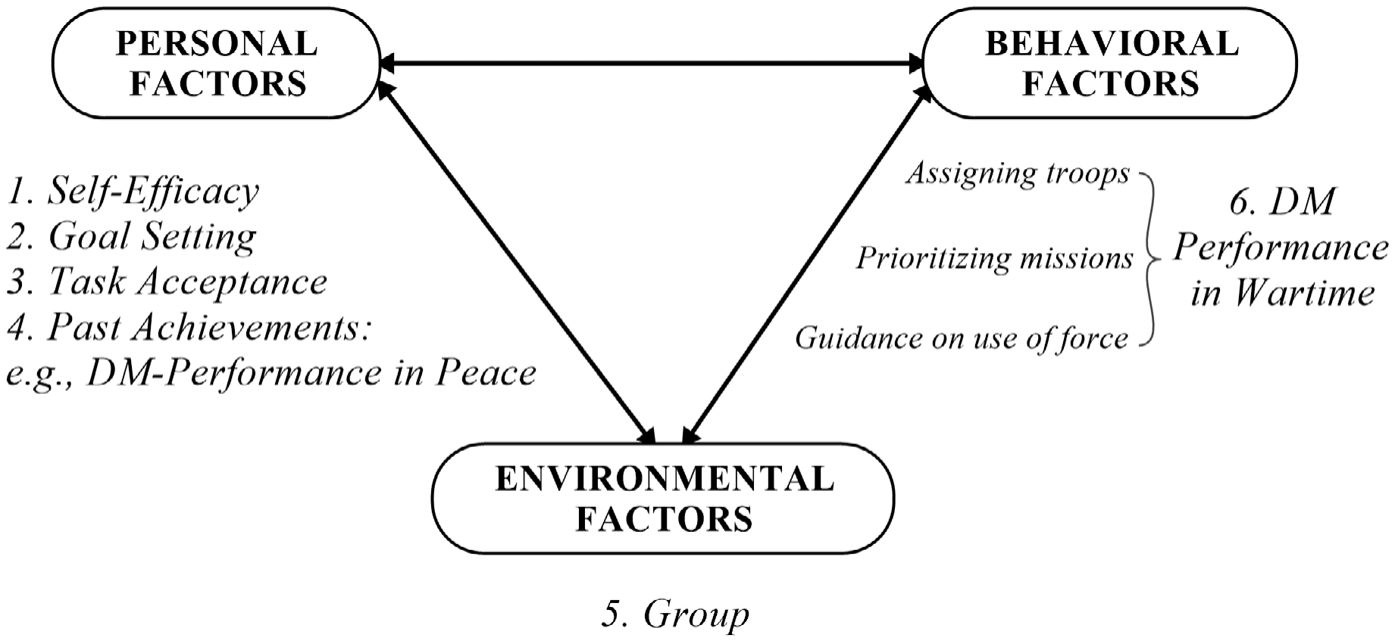
Overview of the study's variables. Adapted from Schunk and DiBenedetto (2021).

At the start of the simulation, the participants were asked to indicate their occupational background by choosing one of two options, “police” or “military”, on a digital questionnaire. The variables are illustrated in Figure 3, highlighting the interplay of factors outlined in SCT.

### 2.6 Data Analysis

The data were analyzed using Jamovi (version 2.3.28) to examine the relationships between peace DM performance, self‐efficacy, task acceptance, goal setting, and war DM performance. A correlation matrix was employed to identify the patterns of relationships between these variables, while path analysis was used to model the direct and indirect effects of the variables on DM performance in war.

### 2.7 Limitations

While this study advances understanding of DM dynamics, several limitations warrant acknowledgment.

#### 2.7.1 Causal Inference

Path analysis identifies plausible causal relationships between self‐efficacy, goal setting, task acceptance, and DM performance but cannot confirm causality absent experimental manipulation.

#### 2.7.2 Scenario Constraints

While the scenario incorporated ecologically valid elements (e.g., transboundary threats and crisis escalation), its controlled design precluded exploration of contextual moderators like organizational culture or hybrid threat evolution.

#### 2.7.3 Measurement Trade‐Offs

While single‐item measures for goal setting and task acceptance may risk oversimplification (Diamantopoulos et al. 2012), we addressed this through several strategies: (a) ensuring the wording of the single‐item VDS measures was clear, (b) utilizing scenario‐anchored VDS response options to provide context, (c) conducting SME cross‐validation to align ratings with the study's intent, and (d) performing a pilot study to assess the effectiveness of the measures and enhance their validity.

#### 2.7.4 SME Subjectivity

Despite rigorous SME selection criteria, their evaluations may reflect confirmation or experience biases, potentially undervaluing unconventional DM.

#### 2.7.5 Sample Scope

The modest sample (*N* = 102), though sufficient for initial modeling, limits generalizability to broader leadership populations. Longitudinal designs tracking these variables across crises would clarify temporal dynamics.

#### 2.7.6 Context Specificity

The wartime focus emphasizes tactical efficacy but neglects interpersonal factors like leadership style or team cohesion, which may dominate in non‐combat operational settings.

#### 2.7.7 Subordinate Exclusion Rationale

Subordinates were omitted because the scenario's isolated command structure rendered their perspectives peripheral to the participants' decisions. Inclusion would have introduced logistical burdens (time, cost) without commensurate theoretical value.

#### 2.7.8 Future Directions

Replications with larger, multisector cohorts, longitudinal frameworks, and expanded variables (e.g., leadership adaptability metrics) could refine these findings for diverse operational contexts.

## 3 Results

Table 1 displays the Pearson correlations for the variables studied. The following two‐tailed correlation coefficients were significant at the 0.05 level: (1) Peace DM performance with war DM performance; (2) Self‐efficacy with goal setting; and (3) Self‐efficacy with task acceptance.

**TABLE 1:** Correlation matrix.

		Peace DMP	War DMP	Self‐efficacy	Task acceptance	Goal setting
Peace DMP	Person *r*	—	—	—	—	—
War DMP	Person *r*	0.42***	—	—	—	—
Self‐efficacy	Person *r*	−0.08	−0.03	—	—	—
Task accept	Person *r*	0.01	0.17	0.20*	—	—
Goal setting	Person *r*	−0.19	0.14	0.35***	0.13	—
Group	Person *r*	0.09	0.03	0.16	0.00	0.01

Abbreviation: DMP, decision‐making performance.

*p < 0.05, ***p < 0.001.

While the correlation coefficients provide insights into the study's research question, a path analysis was conducted to examine the direct and indirect effects among the variables. The final path model exhibited excellent fit to the data, with a non‐significant Chi‐squared (*χ* ^2^) value of 3.22 (5, *N* = 102, *p* = 0.66). Additionally, the Comparative Fit Index (CFI) was 1.00, the Tucker–Lewis Index (TLI) was 1.13, and the standardized root mean square residual (SRMR) was 0.04, all of which indicate a perfect fit to the data. The results of the path analysis are presented in Tables 2 and 3 as standardized and unstandardized scores.

**TABLE 2:** Direct effects and *R*‐squared (*R* ^2^).

Effect	Unstandardized estimate	SE	*β*	*z*	95% CI upper–lower	*R* ^2^
1. Peace DMP	0.39***	0.08	0.44	4.73	0.23 to 0.55	
2. Self‐efficacy	−1.01	1.01	−0.10	−1.00	−2.99 to 0.97	
3. Goal setting	2.34**	0.90	0.23	2.59	0.57 to 4.11	
4. Task acceptance on war DMP	3.05	1.84	0.15	1.66	−0.56 to 6.67	0.27***
1. Group	0.27	0.17	0.16	1.65	−0.05 to 0.60	
2. Peace DMP on self‐efficacy	−0.01	0.01	−0.10	−0.98	−0.03 to 0.01	0.03
1. Self‐efficacy	0.33***	0.10	0.33	3.47	0.15 to 0.52	
2. Task acceptance on goal setting	0.13	0.19	0.07	0.70	−0.24 to 0.50	0.13***
1. Self‐efficacy on task acceptance	0.10*	0.05	0.20	1.96	0.01 to 0.20	0.04*

Abbreviations: CI, confidence intervals; DMP, decision‐making performance; SE, standard error.

*p < 0.05, **p < 0.01, ***p < 0.001, two‐tailed (95% confidence level).

**TABLE 3:** Indirect effects.

Label	Description	Unstandardized estimate	SE	95% CI		*β*	*z*
				Lower	Upper		
IE1	Peace DMP ⇒ Self‐Efficacy ⇒ War DMP	0.01	0.01	−0.02	0.03	0.01	0.69
IE2	Peace DMP ⇒ Self‐Efficacy ⇒ Goal Setting ⇒ War DMP	−0.01	0.01	−0.02	0.01	−0.01	−0.86
IE3	Peace DMP ⇒ Self‐Efficacy ⇒ Task Accept ⇒ War DMP	−0.00	0.00	−0.01	0.00	−0.00	−0.83
IE4	Peace DMP ⇒ Self‐Efficacy ⇒ Task Accept ⇒ Goal Setting ⇒ War DMP	−0.00	0.00	−0.00	0.00	−0.00	−0.57
IE5	Self‐Efficacy ⇒ Goal Setting ⇒ War DMP	0.78*	0.34	0.11	1.45	0.08	2.29
IE6	Self‐Efficacy ⇒ Task Accept ⇒ War DMP	0.30	0.22	−0.12	0.73	0.03	1.40
IE7	Self‐Efficacy ⇒ Task Accept ⇒ Goal Setting ⇒ War DMP	0.03	0.05	−0.07	0.13	0.00	0.62
IE8	Task Accept ⇒ Goal Setting ⇒ War DMP	0.32	0.46	−0.59	1.21	0.02	0.67
IE9	Group ⇒ Self‐Efficacy ⇒ War DMP	−0.27	0.32	−0.90	0.35	−0.02	−0.86
IE10	Group ⇒ Self‐Efficacy ⇒ Goal Setting ⇒ War DMP	0.21	0.15	−0.09	0.35	0.01	1.40
IE11	Group ⇒ Self‐Efficacy ⇒ Task Accept ⇒ War DMP	0.08	0.08	−0.07	0.24	0.00	1.05
IE12	Group ⇒ Self‐Efficacy ⇒ Task Accept ⇒ Goal Setting ⇒ War DMP	0.01	0.01	−0.02	0.04	0.00	0.58

Abbreviations: CI, confidence intervals; DMP, decision‐making performance; SE, standard error.

*p < 0.05, two‐tailed (95% confidence level).

The *R* ^2^ values indicated a significant relationship between the exogenous and endogenous variables and the outcome variable of war DM performance, with a confidence interval (CI) of 0.13–0.42, suggesting that a substantial portion (27%) of the variance in DM performance in wartime can be attributed to the variables in the model. For DM performance measurements in peacetime/war, the SME interrater reliability showed an acceptable intraclass correlation of 0.76.

Furthermore, the *R* ^2^ values revealed a significant relationship between Self‐Efficacy, Task Acceptance, and Goal Setting, with these variables explaining 13% of the variance in Goal Setting, with a CI of 0.03–0.26. This suggests that Self‐Efficacy and Task Acceptance are important drivers of goal setting decisions, and that these variables are closely linked in the model.

Finally, the *R* ^2^ values revealed that Self‐Efficacy accounted for a statistically significant 4% of the variance in Task Acceptance, with a CI of 0.01–0.14. This moderate relationship between Self‐Efficacy and Task Acceptance suggests that while Self‐Efficacy was not the primary driver of Task Acceptance in the model, it did contribute to the explanation of this outcome.

The results revealed statistically significant path coefficients at the 0.05 level, indicating a positive relationship between the following variables:

- Peace DM performance and war DM performance, indicating that performance in peacetime has a direct and positive impact on actual DM performance in war.
- Goal Setting and wartime DM performance, supporting Hypothesis 2, which suggests that goal setting plays a crucial role in determining performance outcomes.
- Self‐Efficacy and Goal Setting, indicating that self‐efficacy has a direct and positive impact on goal setting behaviors.
- Self‐Efficacy and Task Acceptance, suggesting that individuals with higher self‐efficacy are more likely to accept tasks and challenges in war.
- Self‐Efficacy and wartime DM performance, with a positive indirect effect mediated by Goal Setting, supporting Hypothesis 3, which suggests that self‐efficacy influences DM performance in war by shaping goal‐setting behaviors.

**FIGURE 4:**
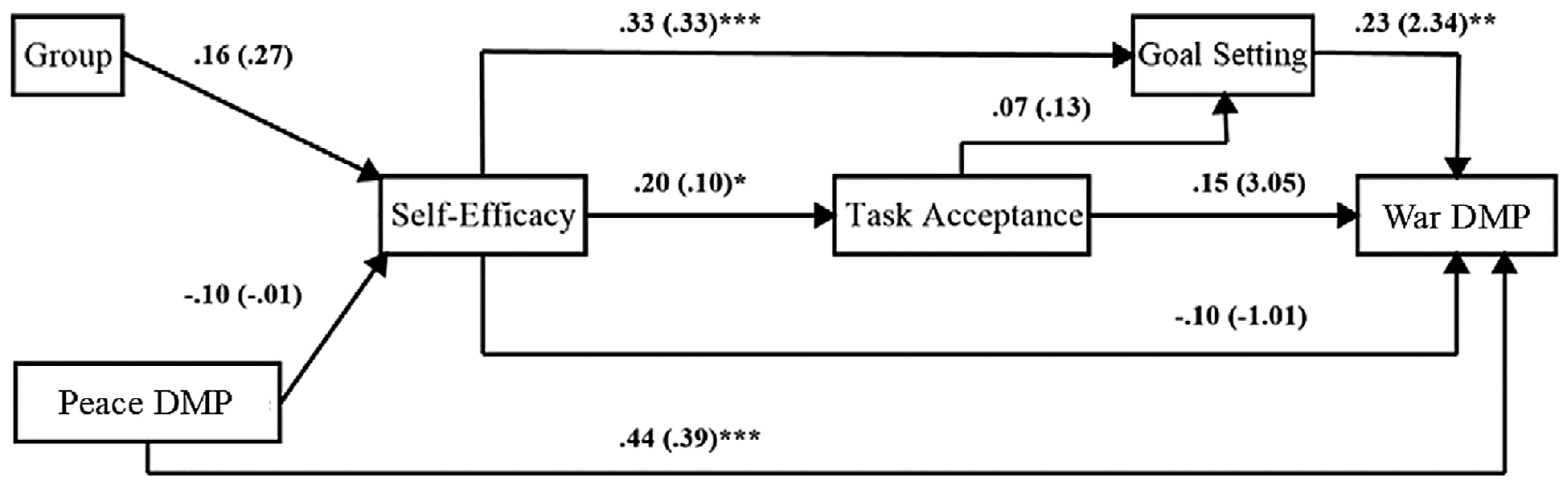
Path analysis output. Path coefficients in the form Beta weights appear outside parentheses. DMP, decision‐making performance, model fit summary: CFI = 1.00, TLI = 1.13, SRMR = 0.04. *p < 0.05, **p < 0.01, ***p < 0.001.

Additionally, the analysis revealed a surprising finding: a non‐significant relationship between Self‐Efficacy and wartime DM performance. This indicates that our Hypothesis 1, which posited a direct link between self‐efficacy and DM performance in wartime, was not supported. The path model is illustrated in Figure 4.

## 4 Discussion

The current study aimed to investigate the relationships between Self‐Efficacy, Task Acceptance, Goal Setting, and Decision‐Making Performance in the context of hybrid warfare. The study's findings have shed new light on the intricate dynamics of DM among senior officials leading from higher headquarters, revealing a complex interplay between these variables. This discussion will delve into the implications of our findings, the theoretical frameworks they support, and the practical considerations for enhancing leadership effectiveness at higher levels of government.

Firstly, while the study's results validated several anticipated relationships, they also challenged previously held assumptions regarding the direct influence of self‐efficacy on DM performance. Specifically, the findings contradicted our Hypothesis 1, indicating that self‐efficacy did not have a direct effect on DM performance in war. This underscores the challenges of operational leadership and emphasizes the need for a more nuanced understanding of the factors that influence DM performance in headquarters environments.

Self‐efficacy has typically been studied in more stable environments, such as traditional organizational settings or controlled laboratory conditions (Schunk and DiBenedetto 2021). The predictable nature of these environments allows for clearer causal relationships between self‐efficacy and DM performance, leading to the assumption that higher self‐efficacy will consistently result in better outcomes (Falco and Summers 2017). However, findings from studies on hybrid warfare suggest that this relationship is less influential in hostile and ambiguous environments (Mattingsdal, Aandal, et al. 2023). In leadership contexts, Bandura and Wood (1989) explain that a variety of external factors, such as incomplete information, unpredictable adversaries, and the need for interoperability among different teams, can weaken the influence of self‐efficacy (p. 805). This highlights the need for a more comprehensive understanding of self‐efficacy in headquarters settings (Rochmawati et al. 2023), where traditional assumptions about what drives performance might not be accurate (Schmutz et al. 2023). Notably, our study found that participants' beliefs in their ability to counter hybrid warfare effectively had a limited impact on their DM performance in war. This implies that other factors, such as strategic thinking and adaptability, may play a more significant role in determining success in crisis response scenarios. By highlighting the limitations of self‐efficacy as a sole predictor of DM performance, our findings contribute to a more nuanced understanding of the complex factors that influence DM at higher levels of government.

Secondly, despite this unexpected finding, the study's results provided valuable insights into the interactions of self‐efficacy, task acceptance, goal setting, and DM performance in wartime. For instance, the significant positive relationship between peacetime DM performance and wartime DM performance aligns with existing literature that emphasizes the importance of expertise in operational settings (Călin 2024). Leaders who have navigated previous crises are likely to draw upon their experiences, leading to improved DM outcomes (Penney et al. 2022). It highlights that skills and self‐regulatory strategies developed in one situation can be applied to different situations, potentially leading to improved performance (Bandura 1997). This transfer is facilitated by the similarity between contexts, the relevance of the skills or knowledge learned in one context to the new context, and the frequency of exposure to the new context.

Our finding indicates that experiences gained during peaceful times can positively impact leaders facing wartime threats. It suggests that crisis response organizations should prioritize experiential training and simulations that replicate real‐world challenges, fostering a culture of continuous learning and adaptation. In the context of countering hybrid warfare from higher headquarters, where the DM environment is dynamic and ambiguous (Mumford and Carlucci 2023), the capacity to learn from past engagements becomes a critical asset for senior leaders tasked with safeguarding national security (Deverell 2021).

Thirdly, our study builds on the existing research on the significance of goal setting in achievement contexts, as highlighted by Marshall and Brown (2004). We reaffirm the finding that performance goals are crucial in achieving optimal outcomes in collaborative crisis response settings, demonstrating a significant and positive relationship between goal setting and wartime DM performance. This is in line with previous research (Criss et al. 2024), providing strong support for Hypothesis 2.

Our findings suggest that the outcome of crisis response operations depends heavily on setting prudent performance goals. When senior leaders prioritize and provide appropriate guidance, subordinates can allocate resources effectively, coordinate efforts among different units, and make informed decisions about the use of force. To enhance unit morale, leaders must approve missions and establish clear, well‐defined performance goals. These goals should be legal, align with broader strategies, and remain adaptable. To achieve this, senior leaders must balance tactical and strategic objectives, choose between collaborative and unilateral approaches, and decide whether to use quantitative or qualitative metrics to measure success.

The significance of goal setting is particularly pronounced in team environments, where ambiguity can lead to confusion and misalignment among individuals and collaborators regarding overarching objectives (Burke et al. 2018). Clear performance goals from higher headquarters are essential for ensuring that all stakeholders are working toward a common objective, and that efforts are focused and coordinated to achieve the desired outcome (Essens et al. 2008). Thus, our findings highlight that the conditions and considerations related to goal setting in higher headquarters, such as the operational context, available resources, and the ambiguity of emerging threats, must be accounted for to ensure effective DM.

The findings related to self‐efficacy offer valuable insights into the role of leadership in crisis response situations, particularly in the context of hybrid warfare. The discovery of a positive relationship between self‐efficacy and DM performance in war mediated by goal setting suggests that leaders who possess a strong belief in their capabilities to manage troops on the ground are more likely to engage in effective decision‐making (Hypothesis 3). This insight highlights the importance of fostering self‐efficacy among leaders situated in higher headquarters, as it can serve as a catalyst for desired mission outcomes. However, the non‐significant direct relationship between self‐efficacy and war DM performance suggests that self‐efficacy may not be a direct determinant of performance outcomes, contrary to what was previously thought (Schunk and DiBenedetto 2021). Instead, it suggests that the effects of self‐efficacy influence DM performance through its impact on goal setting behaviors. For instance, an individual's ability to prioritize tasks and allocate resources, which in turn influences their DM ability. This finding has important implications for leadership development and training programs. Rather than focusing solely on building self‐efficacy, programs should also emphasize the development of goal setting skills and standards.

Moreover, the study's findings raise important questions about the nature of DM in ambiguous environments such as hybrid warfare. The lack of a direct relationship between self‐efficacy and DM performance in wartime suggests that external factors, such as the unpredictability of hybrid warfare and the vicarious nature of leadership in higher headquarters, may moderate this relationship. This nuance underscores the need for senior leaders to develop not only cognitive and emotional competencies but also cultivate team orientation and interoperability skills to facilitate collaborative efforts (Elonheimo 2021). Training programs should therefore incorporate scenarios that challenge leaders to navigate ambiguity and make decisions with incomplete information (Phillips‐Wren and Adya 2020), thereby preparing them for the realities of modern security threats (Shortland et al. 2020).

### 4.1 Practical Implications

As government agencies face the challenges of hybrid warfare, our research highlights the importance of creating team environments that foster effective DM at all echelons of leadership. This involves two key areas:

Higher headquarters must prioritize developing their leaders' DM skills. This can be achieved through mentorship programs, interagency collaboration, and hands‐on learning experiences. These strategies help newer leaders build confidence and improve their goal‐setting skills, making them more effective in their roles. Senior leaders should also focus on clear communication, regular feedback, support and guidance, and continuous learning. By implementing these strategies and principles, higher headquarters can enhance their effectiveness while preparing new leaders to take charge of future operations.

Additionally, headquarters should develop interagency plans that balance the responsibilities of different stakeholders, ensuring that all agree on the standards of adequacy for preparedness and response options. This requires prioritizing long‐term performance goals over short‐term gains and ensuring that all stakeholders are aligned and working toward common objectives. Effective communication of goals is crucial, as it allows headquarters to convey tactical objectives to subordinates, other government agencies, and involved municipalities. For instance, establishing a classified website and an open mobile app could facilitate updates for those affected. This approach ensures both security providers and the public remain informed and can provide feedback on ground‐level developments back to headquarters.

### 4.2 Further Research

While this study contributes to our understanding of DM performance in higher headquarters, several avenues for future research remain. First, research should investigate the role of external factors, such as organizational culture and team dynamics, in moderating the relationships between self‐efficacy, goal setting, and performance. Second, longitudinal studies could explore how these relationships evolve over time, particularly in response to changing operational contexts. Understanding the temporal dynamics of self‐efficacy and performance could provide deeper insights into developing effective leadership in higher headquarters.

Lastly, qualitative studies involving interviews of headquarters leaders engaged in DM regarding hybrid warfare could provide valuable insights into the leadership styles that underpin effective decisions. By capturing the lived experiences of leaders, researchers can identify best practices and nuances that can inform operational leadership development initiatives.

## 5 Conclusion

This study examines the relationships between self‐efficacy, task acceptance, and goal setting, highlighting their interconnected roles in influencing DM performance in hybrid warfare. Characterized by a blend of traditional military tactics and unconventional methods, hybrid warfare presents distinct challenges for leaders in higher headquarters and the teams they manage. Our findings suggest that senior leaders who participated in our study and offer clear strategic guidance are more effective in managing hybrid warfare indirectly through the contributions of their subordinates, underscoring the vital importance of DM skills in leadership at higher levels of government.

Interestingly, our study suggests that while self‐efficacy may not directly boost DM performance in such dynamic environments, it still plays a significant role in fostering goal setting and task acceptance among the leaders participating in our research. This finding challenges the conventional notion that self‐efficacy always leads to better outcomes, instead emphasizing the importance of strategic thinking in enhancing leadership effectiveness at higher echelons of command.

Moreover, participants with high self‐efficacy were more likely to accept challenging tasks and take responsibility for their outcomes, which is particularly valuable in situations characterized by ambiguity. Furthermore, participants who had experienced similar situations in the past demonstrated higher levels of DM performance than their less experienced colleagues, highlighting the significance of commitment and learning from previous experiences in the effective orchestration of interagency efforts.

To enhance a country's defense against hybrid warfare, our research emphasizes the critical role of leadership in facilitating knowledge sharing among security providers and allowing higher headquarters to collaborate effectively. As crisis response increasingly demands interagency cooperation, it is essential to understand the factors that influence DM in headquarters contexts. The insights gained from our research can be instrumental in developing effective leaders and headquarters teams, equipping them with the expertise needed to counter hybrid warfare effectively.

## Author Contributions

Jostein Mattingsdal conducted the data collection, analysis, drafted the initial manuscript, and wrote the final manuscript. Roar Espevik supervised Jostein Mattingsdal during the design phase and contributed to the manuscript's revision, including reviewing and ensuring accuracy.

## Disclosure

The authors have nothing to report.

## Ethics Statement

The study received approval from the Norwegian Center for Research Data (SIKT) and the review board of the Norwegian Defense University College.

## Consent

All participants consented to take part in the study voluntarily and anonymously.

## Data Availability

All data and research materials are accessible to experts in the field upon obtaining explicit permission from the Norwegian Defense University College by reaching out to the authors.

## References

[sjop13120-bib-0001] André, B. , E. Sjøvold , T. Rannestad , and G. I. Ringdal . 2014. “The Impact of Work Culture on Quality of Care in Nursing Homes.” Scandinavian Journal of Caring Sciences 28, no. 3: 449–457. 10.1111/scs.12086. 24117657

[sjop13120-bib-0002] Auglend, R. L. 2015. “The Distribution of Responsibility and Authority Between the Police and the Armed Forces in National Crises.” Tidsskrift for Strafferett 15, no. 3: 316–347. 10.18261/ISSN0809-9537-2015-03-03.

[sjop13120-bib-0003] Bandura, A. 1997. Self‐Efficacy: The Exercise of Control. Freeman.

[sjop13120-bib-0004] Bandura, A. 1999. “Social Cognitive Theory of Personality.” In Handbook of Personality, edited by L. Pervin and O. P. John , 2nd ed., 154–196. Guilford Publications.

[sjop13120-bib-0005] Bandura, A. 2006. “Guide for Constructing Self‐Efficacy Scales.” Self‐Efficacy Beliefs of Adolescents 5, no. 1: 307–337.

[sjop13120-bib-0006] Bandura, A. 2023. Social Cognitive Theory: An Agentic Perspective on Human Nature. John Wiley & Sons. 10.1146/annurev.psych.52.1.1 11148297

[sjop13120-bib-0007] Bandura, A. , and R. Wood . 1989. “Effect of Perceived Controllability and Performance Standards on Self‐Regulation of Complex Decision Making.” Journal of Personality and Social Psychology 56, no. 5: 805–814. 2724068 10.1037//0022-3514.56.5.805

[sjop13120-bib-0008] Bankov, B. 2024. “Hybrid Warfare: How to Escape the Conceptual Gray‐Zone.” Journal of Strategic Security 17, no. 1: 1–23. 10.5038/1944-0472.17.1.2118.

[sjop13120-bib-0009] Bearman, C. , P. Hayes , and M. Thomason . 2023. “Facilitating Teamwork in Emergency Management.” International Journal of Disaster Risk Reduction 97: 103979. 10.1016/j.ijdrr.2023.103979.

[sjop13120-bib-0010] Benight, C. C. , and R. Cieslak . 2011. “Cognitive Factors and Resilience: How Selfefficacy Contributes to Coping With Adversities.” In Resilience and Mental Health: Challenges Across the Lifespan, edited by S. M. Southwick , B. T. Litz , and D. Charney , 45–55. Cambridge University Press.

[sjop13120-bib-0011] Bong, M. 2001. “Between‐ and Within‐Domain Relations of Academic Motivation Among Middle and High School Students.” Journal of Educational Psychology 93, no. 1: 23–34. 10.1037/0022-0663.93.1.23.

[sjop13120-bib-0012] Borch, O. J. , and T. Heier . 2024. Preparing for Hybrid Threats to Security: Collaborative Preparedness and Response. Taylor & Francis.

[sjop13120-bib-0013] Burke, C. S. , M. L. Shuffler , and C. W. Wiese . 2018. “Examining the Behavioral and Structural Characteristics of Team Leadership in Extreme Environments.” Journal of Organizational Behavior 39, no. 6: 716–730. 10.1002/job.2290.

[sjop13120-bib-0014] Călin, C.‐C. 2024. “The Power of Intuition in Decision‐Making Under Operational Stress.” Bulletin of “Carol I” National Defense University 13, no. 2: 79–97. 10.53477/2284-9378-24-21.

[sjop13120-bib-0015] Caliskan, M. , and M. Liégeois . 2021. “The Concept of ‘Hybrid Warfare’ Undermines NATO's Strategic Thinking.” Small Wars & Insurgencies 32, no. 2: 295–319. 10.1080/09592318.2020.1860374.

[sjop13120-bib-0016] Chiriac, C. 2022. “COPD V3. 0 and Implications on the Joint Level.” Bulletin of “Carol I” National Defence University (EN) 11, no. 2: 7–13.

[sjop13120-bib-0017] Criss, C. J. , M. Konrad , S. R. Alber‐Morgan , and M. E. Brock . 2024. “A Systematic Review of Goal Setting and Performance Feedback to Improve Teacher Practice.” Journal of Behavioral Education 33, no. 2: 275–296.

[sjop13120-bib-0018] Dahlstrom, N. , S. Dekker , R. van Winsen , and J. Nyce . 2009. “Fidelity and Validity of Simulator Training.” Theoretical Issues in Ergonomics Science 10, no. 4: 305–314. 10.1080/14639220802368864.

[sjop13120-bib-0019] Deverell, E. 2021. “Learning and Crisis.” In Oxford Research Encyclopedias, edited by E. Hannah and E. M. Kuhonta . Oxford University Press. 10.1093/acrefore/9780190228637.013.1558.

[sjop13120-bib-0020] Diamantopoulos, A. , M. Sarstedt , C. Fuchs , P. Wilczynski , and S. Kaiser . 2012 2012/05/01. “Guidelines for Choosing Between Multi‐Item and Single‐Item Scales for Construct Measurement: A Predictive Validity Perspective.” Journal of the Academy of Marketing Science 40, no. 3: 434–449. 10.1007/s11747-011-0300-3.

[sjop13120-bib-0021] Dwyer, L. P. 2019. “Leadership Self‐Efficacy: Review and Leader Development Implications.” Journal of Management Development 38, no. 8: 637–650.

[sjop13120-bib-0022] Elonheimo, T. 2021. “Comprehensive Security Approach in Response to Russian Hybrid Warfare.” Strategic Studies Quarterly 15, no. 3: 113–137.

[sjop13120-bib-0023] Essens, P. J. , A. L. Vogelaar , J. J. Mylle , et al. 2008. “Team Effectiveness in Complex Settings.” In Team Effectiveness in Complex Organizations, edited by E. Salas , G. F. Goodwin , and C. S. Burke , 327–354. Routledge.

[sjop13120-bib-0024] Falco, L. D. , and J. J. Summers . 2017. “Improving Career Decision Self‐Efficacy and STEM Self‐Efficacy in High School Girls.” Journal of Career Development 46, no. 1: 62–76. 10.1177/0894845317721651.

[sjop13120-bib-0025] Fisher, G. G. , R. A. Matthews , and A. M. Gibbons . 2016. “Developing and Investigating the Use of Single‐Item Measures in Organizational Research.” Journal of Occupational Health Psychology 21, no. 1: 3–23. 10.1037/a0039139. 25894198

[sjop13120-bib-0026] Fosse, T. H. , R. Buch , R. Säfvenbom , and M. Martinussen . 2015. “The Impact of Personality and Self‐Efficacy on Academic and Military Performance: The Mediating Role of Self‐Efficacy.” Journal of Military Studies 6, no. 1: 47–65.

[sjop13120-bib-0027] Galesic, M. , and M. Bosnjak . 2009. “Effects of Questionnaire Length on Participation and Indicators of Response Quality in a Web Survey.” Public Opinion Quarterly 73, no. 2: 349–360. 10.1093/poq/nfp031.

[sjop13120-bib-0029] Gilbert, S. L. , and E. K. Kelloway . 2014. “Leadership.” In The Oxford Handbook of Work Engagement, Motivation, and Self‐Determination Theory, edited by M. Gagné , 181–198. Oxford University Press.

[sjop13120-bib-0030] Gray, C. S. 2015. Tactical Operations for Strategic Effect: The Challenge of Currency Conversion. JSOU Press.

[sjop13120-bib-0031] Grier, R. A. 2012. “Military Cognitive Readiness at the Operational and Strategic Levels.” Journal of Cognitive Engineering and Decision Making 6, no. 4: 358–392. 10.1177/1555343412444606.

[sjop13120-bib-0032] Hettiachchi, D. , S. Wijenayake , S. Hosio , V. Kostakos , and J. Goncalves . 2020. “How Context Influences Cross‐Device Task Acceptance in Crowd Work.” In *Proceedings of the AAAI Conference on Human Computation and Crowdsourcing*.

[sjop13120-bib-0033] Hollenbeck, J. R. , and H. J. Klein . 1987. “Goal Commitment and the Goal‐Setting Process: Problems, Prospects, and Proposals for Future Research.” Journal of Applied Psychology 72, no. 2: 212–220.

[sjop13120-bib-0034] Huang, C. 2016. “Achievement Goals and Self‐Efficacy: A Meta‐Analysis.” Educational Research Review 19: 119–137. 10.1016/j.edurev.2016.07.002.

[sjop13120-bib-0035] Ibrahim, A. U. , and C. O. Daniel . 2019. “Impact of Leadership on Organisational Performance.” International Journal of Business Management and Social Research 6, no. 2: 367–374. 10.18801/ijbmsr.060219.39.

[sjop13120-bib-1001] Jervis, R. 2017. How Statesmen Think: The Psychology of International Politics. Princeton University Press.

[sjop13120-bib-0036] Johnsen, B. H. , R. Espevik , E.‐R. Saus , S. Sanden , O. K. Olsen , and S. W. Hystad . 2017. “Hardiness as a Moderator and Motivation for Operational Duties as Mediator.” Journal of Police and Criminal Psychology 32: 331–339.

[sjop13120-bib-0037] Judge, T. A. , C. L. Jackson , J. C. Shaw , B. A. Scott , and B. L. Rich . 2007. “Self‐Efficacy and Work‐Related Performance.” Journal of Applied Psychology 92, no. 1: 107. 17227155 10.1037/0021-9010.92.1.107

[sjop13120-bib-0038] Kleingeld, A. , H. van Mierlo , and L. Arends . 2011. “The Effect of Goal Setting on Group Performance.” Journal of Applied Psychology 96, no. 6: 1289–1304. 10.1037/a0024315. 21744940

[sjop13120-bib-0039] Locke, E. A. 2023. “Motivate Employee Performance Through Goal Setting.” In Handbook of Principles of Organizational Behavior, edited by E. A. Locke and C. Pearce , 3nd ed. John Wiley & Sons.

[sjop13120-bib-0041] Locke, E. A. , and G. P. Latham . 2013. “Goal Setting Theory: The Current State.” In New Developments in Goal Setting and Task Performance, 623–630. Routledge.

[sjop13120-bib-0043] Marchau, V. A. , W. E. Walker , P. J. Bloemen , and S. W. Popper . 2019. Decision Making Under Deep Uncertainty: From Theory to Practice. Springer Nature.

[sjop13120-bib-0044] Margolis, H. , and P. P. McCabe . 2006. “Improving Self‐Efficacy and Motivation.” Intervention in School and Clinic 41, no. 4: 218–227. 10.1177/10534512060410040401.

[sjop13120-bib-0045] Marshall, M. A. , and J. D. Brown . 2004. “Expectations and Realizations.” Motivation and Emotion 28, no. 4: 347–361. 10.1007/s11031-004-2388-y.

[sjop13120-bib-0046] Masal, D. , and R. Vogel . 2016. “Leadership, Use of Performance Information, and Job Satisfaction.” International Public Management Journal 19, no. 2: 208–234. 10.1080/10967494.2016.1143422.

[sjop13120-bib-0047] Mata, Á. N. d. S. , K. P. M. de Azevedo , L. P. Braga , et al. 2021. “Training in Communication Skills for Self‐Efficacy of Health Professionals.” Human Resources for Health 19: 1–9. 33676515 10.1186/s12960-021-00574-3 PMC7937280

[sjop13120-bib-0048] Mattingsdal, J. , J. Aandal , B. H. Johnsen , and R. Espevik . 2023. “From Peacetime to War.” Frontiers in Psychology 14: 1238760. 10.3389/fpsyg.2023.1238760. 38187420 PMC10766834

[sjop13120-bib-0049] Mattingsdal, J. , R. Espevik , B. H. Johnsen , and S. Hystad . 2024. “Exploring Why Police and Military Commanders Do What They Do.” Armed Forces & Society 50, no. 4: 1218–1244. 10.1177/0095327x231160711.

[sjop13120-bib-0050] Mattingsdal, J. , B. H. Johnsen , and R. Espevik . 2023. “Effect of Changing Threat Conditions on Police and Military Commanders' Preferences for Urgent and Offensive Actions.” Military Psychology 37, no. 1: 1–17. 10.1080/08995605.2023.2277609. 37921694 PMC11649221

[sjop13120-bib-0051] McLarnon, M. J. , M. G. Rothstein , and G. A. King . 2021. “Resiliency to Adversity in Military Personnel: The Role of Self‐Regulation.” Military Psychology 33, no. 2: 104–114. 38536349 10.1080/08995605.2021.1897492 PMC10013496

[sjop13120-bib-0052] Moritz, S. E. , D. L. Feltz , K. R. Fahrbach , and D. E. Mack . 2000. “The Relation of Self‐Efficacy Measures to Sport Performance.” Research Quarterly for Exercise and Sport 71, no. 3: 280–294. 10999265 10.1080/02701367.2000.10608908

[sjop13120-bib-0053] Mumford, A. , and P. Carlucci . 2023. “Hybrid Warfare: The Continuation of Ambiguity by Other Means.” European Journal of International Security 8, no. 2: 192–206. 10.1017/eis.2022.19.

[sjop13120-bib-0054] NATO . 2019. Allied Joint Doctrine for the Planning of Operations (AJP5). NATO Standardization Office (NSO). https://www.coemed.org/files/stanags/01_AJP/AJP‐5_EDA_V2_E_2526.pdf.

[sjop13120-bib-0055] Nordmo, M. , H. O. Sørlie , O. C. Lang‐Ree , and T. H. Fosse . 2022. “Decomposing the Effect of Hardiness in Military Leadership Selection and the Mediating Role of Self‐Efficacy Beliefs.” Military Psychology 34, no. 6: 697–705. 10.1080/08995605.2022.2054658. 38536370 PMC10013544

[sjop13120-bib-0056] Østensen, Å. G. , and T. Bukkvoll . 2018. “Russian Use of Private Military and Security Companies‐the Implications for European and Norwegian Security.” FFI‐Rapport.

[sjop13120-bib-0057] Paglis, L. L. 2010. “Leadership Self‐Efficacy: Research Findings and Practical Applications.” Journal of Management Development 29, no. 9: 771–782.

[sjop13120-bib-0058] Penney, G. , D. Launder , J. Cuthbertson , and M. B. Thompson . 2022 2022/08/01. “Threat Assessment, Sense Making, and Critical Decision‐Making in Police, Military, Ambulance, and Fire Services.” Cognition, Technology & Work 24, no. 3: 423–439. 10.1007/s10111-022-00694-3.

[sjop13120-bib-0059] Phillips‐Wren, G. , and M. Adya . 2020. “Decision Making Under Stress.” Journal of Decision Systems 29, no. suppl 1: 213–225. 10.1080/12460125.2020.1768680.

[sjop13120-bib-0060] Rochmawati, L. , Fatmawati , and M. M. Sukma . 2023. “Motivation, Anxiety, and Self‐Efficacy in Learning Aviation English.” Asian‐Pacific Journal of Second and Foreign Language Education 8, no. 1: 40. 10.1186/s40862-023-00212-6.

[sjop13120-bib-0061] Rutherford, A. , and K. J. Meier . 2015. “Managerial Goals in a Performance‐Driven System.” Public Administration 93, no. 1: 17–33.

[sjop13120-bib-0062] Schmutz, J. B. , N. Bienefeld , M. T. Maynard , and R. Rico . 2023. “Exceeding the Ordinary.” Group & Organization Management 48, no. 2: 581–628. 10.1177/10596011221150756. 37082422 PMC10108401

[sjop13120-bib-0063] Schunk, D. H. , and M. K. DiBenedetto . 2020. “Motivation and Social Cognitive Theory.” Contemporary Educational Psychology 60: 101832.

[sjop13120-bib-0064] Schunk, D. H. , and M. K. DiBenedetto . 2021. “Self‐Efficacy and Human Motivation.” In Advances in Motivation Science, vol. 8, 153–179. Elsevier.

[sjop13120-bib-0065] Seijts, G. H. , and G. P. Latham . 2012. “Knowing When to Set Learning Versus Performance Goals.” Organizational Dynamics 41, no. 1: 1–6.

[sjop13120-bib-0066] Senko, C. , and B. Dawson . 2017. “Performance‐Approach Goal Effects Depend on How They Are Defined.” Journal of Educational Psychology 109, no. 4: 574–598. 10.1037/edu0000160.

[sjop13120-bib-0067] Shortland, N. , L. Alison , and L. Thompson . 2020. “Military Maximizers.” Personality and Individual Differences 163: 110051. 10.1016/j.paid.2020.110051.

[sjop13120-bib-0068] Shortland, N. D. , L. J. Alison , and J. M. Moran . 2019. Conflict: How Soldiers Make Impossible Decisions. Oxford University Press.

[sjop13120-bib-0069] Stajkovic, A. , and K. Sergent . 2019. Social Cognitive Theory, Obo in Management. Oxford Bibliographies. 10.1093/OBO/9780199846740-0169.

[sjop13120-bib-0070] Stajkovic, A. D. , D. Lee , and A. J. Nyberg . 2009. “Collective Efficacy, Group Potency, and Group Performance.” Journal of Applied Psychology 94, no. 3: 814. 19450017 10.1037/a0015659

[sjop13120-bib-0071] Stein, J. 2023. “Perceiving Threat Cognition, Emotion, and Judgment.” In The Oxford Handbook of Political Psychology, edited by L. Huddy , D. Sears , J. Levy , and J. Jerit . Oxford University Press, Incorporated.

[sjop13120-bib-0072] Storberget, K. , K. Bruusgaard , S. Bjørbæk , and C. Chamrer . 2023. NOU 2023: 14 Forsvarskommisjonen av 2021. Norwegian Ministry of Defence. https://www.regjeringen.no/no/dokumenter/nou‐2023‐14/id2974821/.

[sjop13120-bib-0073] Swann, C. , S. Rosenbaum , A. Lawrence , S. A. Vella , D. McEwan , and P. Ekkekakis . 2021. “Updating Goal‐Setting Theory in Physical Activity Promotion.” Health Psychology Review 15, no. 1: 34–50. 31900043 10.1080/17437199.2019.1706616

[sjop13120-bib-0074] Taylor, M. K. , K. E. Stanfill , G. A. Padilla , et al. 2011. “Effect of Psychological Skills Training During Military Survival School.” Military Medicine 176, no. 12: 1362–1368. 10.7205/milmed-d-11-00149. 22338349

[sjop13120-bib-0075] Wang, S. , and I. C. Hsu . 2014. “The Effect of Role Ambiguity on Task Performance Through Self‐Efficacy—A Contingency Perspective.” IEEE Transactions on Engineering Management 61, no. 4: 681–689. 10.1109/TEM.2014.2356341.

[sjop13120-bib-0076] Weaver, J. M. 2021. “Joint Warfare Centre (JWC).” In NATO in Contemporary Times: Purpose, Relevance, Future, 149–163. Springer.

[sjop13120-bib-0077] Wegge, N. 2023. Sikkerhetspolitikk og Militærmakt i Arktis. Cappelen Damm Akademisk.

[sjop13120-bib-0078] Weissmann, M. , N. Nilsson , P. Thunholm , and B. r. Palmertz . 2021. Hybrid Warfare: Security and Asymmetric Conflict in International Relations. I.B. Tauris.

[sjop13120-bib-0079] Wilson, J. , and L. B. Cadin . 2024. “Contesting Russia Report.” Commission on Security and Cooperation in Europe. https://www.csce.gov/wp‐content/uploads/2024/09/Contesting‐Russia‐Report‐2.pdf.

[sjop13120-bib-0080] Wood, R. , and A. Bandura . 1989. “Social Cognitive Theory of Organizational Management.” Academy of Management Review 14, no. 3: 361–384.

[sjop13120-bib-0081] Wood, R. , A. Bandura , and T. Bailey . 1990. “Mechanisms Governing Organizational Performance in Complex Decision‐Making Environments.” Organizational Behavior and Human Decision Processes 46, no. 2: 181–201.

[sjop13120-bib-0083] Zhang, P. , D. Liu , and S. Lyu . 2023. “Leadership Mobility and Target Adaptation.” Public Performance & Management Review 47, no. 1: 204–231. 10.1080/15309576.2023.2259367.

[sjop13120-bib-0084] Zimmer‐Gembeck, M. J. 2021. “Coping Flexibility: Variability, Fit and Associations With Efficacy, Emotion Regulation, Decentering and Responses to Stress.” Stress and Health 37, no. 5: 848–861. 33720506 10.1002/smi.3043

